# The proteome of granulovacuolar degeneration and neurofibrillary tangles in Alzheimer’s disease

**DOI:** 10.1007/s00401-020-02261-4

**Published:** 2021-01-25

**Authors:** David C. Hondius, Frank Koopmans, Conny Leistner, Débora Pita-Illobre, Regina M. Peferoen-Baert, Fenna Marbus, Iryna Paliukhovich, Ka Wan Li, Annemieke J. M. Rozemuller, Jeroen J. M. Hoozemans, August B. Smit

**Affiliations:** 1 Department of Pathology, Amsterdam Neuroscience, Amsterdam University Medical Centers, Location VUmc, PO Box 7057, Amsterdam, 1007 MB The Netherlands; 2 Department of Molecular and Cellular Neurobiology, Center for Neurogenomics and Cognitive Research, Amsterdam Neuroscience, VU University Amsterdam, Amsterdam, The Netherlands

**Keywords:** Alzheimer´s disease, Granulovacuolar degeneration, Tau, Neurofibrillary tangles, Proteomics, Neuropathology

## Abstract

Granulovacuolar degeneration (GVD) is a common feature in Alzheimer’s disease (AD). The occurrence of GVD is closely associated with that of neurofibrillary tangles (NFTs) and GVD is even considered to be a pre-NFT stage in the disease process of AD. Currently, the composition of GVD bodies, the mechanisms associated with GVD and how GVD exactly relates to NFTs is not well understood. By combining immunohistochemistry (IHC) and laser microdissection (LMD) we isolated neurons with GVD and those bearing tangles separately from human post-mortem AD hippocampus (*n* = 12) using their typical markers casein kinase (CK)1δ and phosphorylated tau (AT8). Control neurons were isolated from cognitively healthy cases (*n* = 12). 3000 neurons per sample were used for proteome analysis by label free LC–MS/MS. In total 2596 proteins were quantified across samples and a significant change in abundance of 115 proteins in GVD and 197 in tangle bearing neurons was observed compared to control neurons. With IHC the presence of PPIA, TOMM34, HSP70, CHMP1A, TPPP and VXN was confirmed in GVD containing neurons. We found multiple proteins localizing specifically to the GVD bodies, with VXN and TOMM34 being the most prominent new protein markers for GVD bodies. In general, protein groups related to protein folding, proteasomal function, the endolysosomal pathway, microtubule and cytoskeletal related function, RNA processing and glycolysis were found to be changed in GVD neurons. In addition to these protein groups, tangle bearing neurons show a decrease in ribosomal proteins, as well as in various proteins related to protein folding. This study, for the first time, provides a comprehensive human based quantitative assessment of protein abundances in GVD and tangle bearing neurons. In line with previous functional data showing that tau pathology induces GVD, our data support the model that GVD is part of a pre-NFT stage representing a phase in which proteostasis and cellular homeostasis is disrupted. Elucidating the molecular mechanisms and cellular processes affected in GVD and its relation to the presence of tau pathology is highly relevant for the identification of new drug targets for therapy.

**Supplementary Information:**

The online version contains supplementary material available at 10.1007/s00401-020-02261-4.

## Introduction

Besides amyloid plaques and neurofibrillary tangles (NFTs), AD pathology is commonly featured by the presence of granulovacuolar degeneration (GVD) in neurons. GVD appears as rimmed vacuoles that are up to 5 µm in diameter, harbouring a dense core, and are visible in a standard haematoxylin stain. These GVD bodies initially emerge predominantly in hippocampal pyramidal neurons in the hippocampal regions CA1, CA2 and subiculum. In later stages of AD, GVD presents in other brain regions, such as the temporal lobe, the hypothalamus and the amygdala [67]. Various studies [19, 23, 29, 45, 76] have pointed at an association between GVD and the onset of tau pathology. GVD is observed in non-demented cases that have a low Braak stage for tau pathology and is considered to be part of pre-clinical AD or normal aging. A gradual increase in the fraction of neurons with GVD is observed with the progression of AD and correlates with the increase of tau pathology in these regions [29, 46]. GVD is also present in Down’s syndrome and primary tauopathies [44, 61, 64], as well as frontotemporal dementia cases with C9orf72 mutations and alpha synucleinopathies, such as Parkinson’s disease (PD), Lewy body dementia (LBD) and multiple system atrophy (MSA) [18, 37, 57]. This indicates that GVD is not exclusively associated with the aggregation of tau, but occurs more broadly in neurodegenerative diseases affected by intracellular protein aggregates.

The cause or function of GVD in neurodegeneration is elusive. GVD bodies are known to contain proteins involved in the unfolded protein response (pPERK, pIRE1 and peIF2α) [19, 30], the endocytosis pathway (CHMP2B) [75] and late stage autophagy (LAMP1 and to some extent cathepsin-D) [10] representing mechanisms that are thought to manage an overload of misfolded proteins. Also necrosome activation markers were detected in GVD bodies as a possible cause of neuronal loss by delayed necroptosis [31]. In addition, various kinases are present in GVD including the protein kinases casein kinase 1 (CK1) α, δ and ε, and are commonly used markers to visualize GVD bodies [12]. These protein kinases are involved in numerous cellular processes and are capable of tau phosphorylation [28, 62]. The increased presence of the aforementioned proteins in GVD affected neurons points towards activation or malfunction of various cellular mechanisms related to stress responses, protein folding and protein degradation.

For various reasons it is hypothesized that GVD in AD brain is associated with an early stage in neurofibrillary tangle formation [29]. GVD frequently occurs in so-called pre-tangle neurons defined as neurons with diffuse hyperphosporylated tau (pTau) immunoreactivity [19]. GVD bodies are present in neurons with markers of early tangle formation including truncated tau, early pTau epitopes and high 4-repeat tau presence, but is not associated with late pTau markers like AT100 and AT270 and the presence of 3-repeat tau [15, 45]. Also, kinases that are known to phosphorylate tau are present in GVD containing neurons [6, 12, 34, 36, 43]. Although GVD is closely associated with markers for cellular stress and early stages of tau phosphorylation, it remains unclear whether GVD is a separate pathological process or part of a pre-stage of the neurofibrillary tangle. Currently, no comprehensive human based quantitative assessment of protein abundances on GVD and tangle bearing neurons has been performed which could support this hypothesis. In addition, elucidating the molecular mechanisms and cellular processes affected in GVD and its relation to tangle pathology is highly relevant for the identification of new drug targets for future therapy.

To increase our insight in the role of GVD in the pathogenesis of AD, we have applied a combination of immunohistochemistry (IHC), cellular-resolution laser microdissection (LMD) and subsequently a mass spectrometry (LC–MS/MS) driven proteomics analysis. We isolated and analysed the proteome of separate populations of hippocampal pyramidal neurons, namely; (1) neurons with CK1δ positive GVD bodies, (2) neurons with pTau positive tangles, and (3) neurons negative for both CK1δ and pTau. This approach provides a highly sensitive, unbiased quantification of proteins at cellular resolution. Specifically, we were able to identify prominent cellular mechanisms involved in GVD, and show the coherence between GVD affected neurons and those with neurofibrillary tangles, supporting a model in which GVD is part of an early phase in tau pathology.

## Material and methods

### Case selection

Post mortem brain tissue was obtained from the Netherlands Brain Bank (NBB), Netherlands Institute for Neuroscience (NIN), Amsterdam. All brain tissue was collected from donors with written informed consent for brain autopsy and the use of brain tissue and clinical information for research purposes. The brain donor program of the NBB was approved by the local medical ethics committee of the VU university medical center (Ref#2009/148). Brain tissue was selected based on clinical and neuropathological reports. Control cases used for proteomics had very little to no abnormalities including Aβ, tau, GVD, α-synuclein or p62 pathology in the hippocampus and were cognitively healthy. AD cases used for proteomics were typical AD cases with severe tau pathology in the hippocampus, but no other comorbidities in the hippocampal region. Per group 12 cases were used for LC/MS–MS analysis which are listed in Table 1. Samples were analysed in two separate batches which consisted of 6 cases per group, marked by batch “A” and batch “B” in Table 1. Cases used for validation are listed in Table S1. AD pathology present as Aβ deposits, neurofibrillary tangles and neuritic plaques was staged [2, 3, 39] and also the ABC score is provided [2, 3, 39, 40, 66].

**Table 1:** Cases used for mass spectrometry analysis

Batch	Case	Braak tau	Braak Aβ	ABC score	Gender	Age at death	PMD (hh:mm)	APOE genotype
1	Control 1	0	0	A0B0C0	M	56	9:15	ε3 ε4
1	Control 2	0	0	A0B0C0	M	74	8:05	ε3 ε3
1	Control 3	0	0	A0B0C0	M	49	6:15	ε2 ε3
1	Control 4	0	0	A0B0C0	M	76	6:45	ε3 ε3
1	Control 5	0	0	A0B0C0	F	62	5:00	ND
1	Control 6	0	0	A0B0C0	F	64	5:40	ε2 ε3
2	Control 7	1	A	A1B1C0	F	71	7:10	ε2 ε3
2	Control 8	1	0	A0B1C0	M	64	8:25	ε3 ε3
2	Control 9	0	0	A0B0C0	M	51	7:45	ε3 ε4
2	Control 10	1	0	A0B1C0	M	78	17:40	ε3 ε3
2	Control 11	1	0	A0B1C0	F	50	4:10	ND
2	Control 12	1	B	A1B1C0	M	80	3:18	ND
1	AD 1	6	B	A2B3C3	M	65	5:40	ε3 ε3
1	AD 2	5	C	A3B3C3	F	77	7:00	ε3 ε3
1	AD 3	5	C	A3B3C3	F	89	10:20	ε3 ε3
1	AD 4	5	C	A3B3C3	F	78	4:35	ε3 ε4
1	AD 5	6	C	A3B3C3	F	64	5:30	ε3 ε4
1	AD 6	5	C	A3B3C3	F	82	6:00	ε4 ε4
2	AD 7	5	C	A3B3C3	M	84	4:50	ε3 ε4
2	AD 8	5	C	A3B3C3	F	78	8:25	ND
2	AD 9	5	C	A3B3C3	F	81	6:10	ND
2	AD 10	5	C	A3B3C3	M	65	6:50	ε3 ε4
2	AD 11	6	C	A3B3C3	F	65	6:40	ε3 ε3
2	AD 12	6	C	A3B3C3	M	62	8:15	ND

*AD* Alzheimer’s disease, *PMD* post-mortem delay, *M* male, *F* female, *ND* not determined

### Fast immunohistochemistry for laser microdissection (LMD)

Sample preparation for mass spectrometry was performed as previously described [17]. Briefly, Sections (10 µm) of fresh-frozen human hippocampus were mounted on PEN-membrane slides (Leica Wetzlar, Germany), air-dried and fixed in 100% ethanol for 1 min. After air-drying the tissue was wetted with sterile phosphate-buffered saline (PBS) pH 7.4.

Tissue sections were immunostained for CK1δ (Santa Cruz, sc-55553) at a 1:25 dilution in PBS pH 7.4 to detect GVD bodies, neurofibrillary tangles were detected using a pTau (AT8) antibody (Pierce Biotechnology, Rockford, IL, USA) at a 1:100 dilution in PBS pH 7.4. Both were incubated for 20 min and then briefly washed in sterile PBS pH 7.4 (3 × 30 s). Next, HRP labelled rabbit anti-mouse (DAKO, Glostrup, Denmark) was applied at a 1:100 dilution in sterile PBS and incubated for 15 min at RT and again briefly washed in sterile PBS pH 7.4 (3 × 30 s). Freshly prepared 3,3′ diaminobenzidine (DAB) solution (DAKO) was applied and left to incubate for 5 min to visualize antibody binding. Sections were thoroughly washed in ultra-pure H_2_O and incubated with 1% (w/v) toluidine blue (Fluka Analytical, Buchs, Switzerland) in ultrapure H_2_O for 1 min as a counterstain. The toluidine blue counterstain allowed identification of control neurons in which no immunoreactivity was present. Sections were then washed in ultra-pure H_2_O twice for 1 min and twice in 100% ethanol for 1 min and air dried.

### Isolation of individual neurons using LMD

**Fig. 1:**
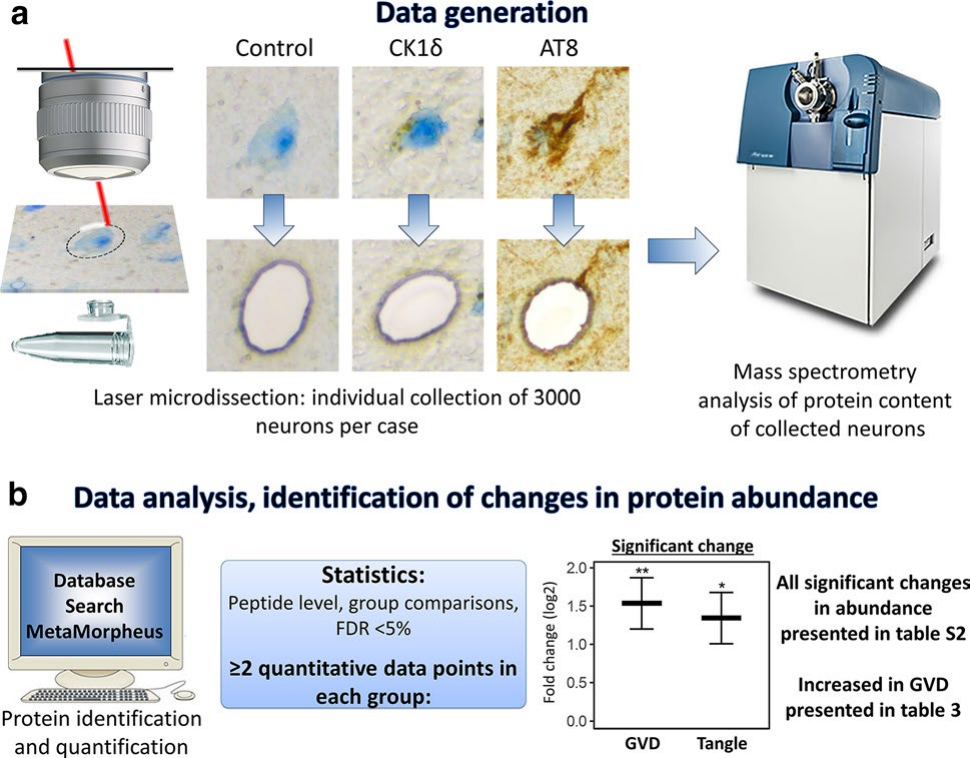
Analysis workflow. Individual neurons were isolated from postmortem human hippocampal brain tissue by laser microdissection (LMD). Using immunohistochemistry three populations of neurons were visualized and isolated with high accuracy: 1. Control neurons, from cognitively healthy control cases that were negative for CK1δ and pTau (AT8) immunoreactivity, 2. granulovacuolar degeneration (GVD) bearing neurons, as recognised by the typical CK1δ positive granular staining pattern, and 3. pTau positive neurons, mostly tangle-bearing neurons, were isolated from AD cases. For each neuronal population 3000 individual neurons were isolated per case. The protein content of each sample was then analysed using mass spectrometry (**a**). Peptide level statistics were applied to identify proteins that are differentially expressed in neurons with GVD and neurons with pTau compared to control neurons (**b**). All resulting proteins are listed in table S2. The subset of proteins which are increased in GVD are listed in Table 3

LMD was performed using a Leica LMD6500 system (Leica, Wetzlar, Germany). Control neurons were isolated from the CA1 and subiculum region in the hippocampus from healthy control cases that were negative for AT8 immunoreactivity and had no GVD marked by granular CK1δ staining. Neurons with GVD, marked by CK1δ staining, and tangle bearing neurons marked by AT8 positivity were isolated from the same region from AD cases (Fig. 1).

Per sample approximately 3.000 individual neurons were collected into caps of tubes (Sapphire PCR/Tubes, 0.5 ml Greiner Bio-One, Solingen, Germany) containing 30 µl M-PER lysis buffer (Thermo Scientific) supplemented with reducing SDS sample buffer (Thermo Scientific) for batch one. For the second batch approximately 3000 individual neurons were collected into adhesive caps (Carl Zeiss, Germany) and 30 µl M-PER lysis buffer (Thermo Scientific) supplemented with reducing SDS sample buffer (Thermo Scientific) was added later. Micro-dissected tissue was stored at − 80 °C until further use.

### Protein in-gel digestion

Micro-dissected tissue lysates were incubated at 95 °C for 5 min to denature the proteins followed by incubation with 50 mM iodoacetamide for 30 min at room temperature (RT) in the dark. Samples were loaded onto a 10% acrylamide gel, of 1 mm thickness. Gels were composed of 10% acrylamide, 0.375 M Tris–HCl (pH 8.8), ultra-pure H_2_O, 0.1% (w/v) APS and 6 µL N,N,N′,N′-tetramethylethylene-diamine (TEMED) per gel. Proteins were allowed to migrate into the gel by electrophoresis (150 V), until the sample progressed in the gel for a length of approximately 8–10 mm.

Gels were fixed overnight in a solution containing 50% (v/v) ethanol and 3% (v/v) phosphoric acid in H_2_O at RT and stained with Colloidal Coomassie Blue (34% (v/v) methanol, 3% (v/v) phosphoric acid, 15% (w/v) ammonium Sulphate, and 0.1% (w/v) Coomassie brilliant blue G-250, for 1 h while shaking. The gel was washed in ultra-pure water under gentle agitation for several hours to reduce background staining. Per sample the part of the gel containing the proteins was separated and cut into blocks of approximately 1 mm^3^ and collected in an Eppendorf tube. Gel fragments were destained in ultrapure water with 50 mM NH_4_HCO_3_ and 50% (v/v) acetonitrile overnight. Gel fragments were dehydrated using acetonitrile for 20 min and dried for 30 min using a speedvac. The gel parts were rehydrated in 70 µl of ultra-pure water containing 50 mM NH_4_HCO_3_ and 10 µg/ml trypsin (sequence grade; Promega) and incubated overnight at 37 °C to facilitate digestion of the proteins. Peptides were extracted twice with a solution containing 0.1% (v/v) trifluoroacetic acid and 50% (v/v) acetonitrile for 20 min. The samples were dried using a speedvac and stored at − 20 °C until further analysis.

### Mass spectrometry analysis

Peptides of the individual sample fractions were dissolved in 12 µL of 0.1% (v/v) acetic acid. In the first set, the sample was loaded onto an Ultimate 3000 LC system (Dionex, Thermo Scientific). Peptides were trapped on a 5 mm Pepmap100 C18 column (Dionex) and fractionated on a 200 mm Alltima C18 column (100 μm id, 3 μm particle size). In the second set, the sample was loaded onto a nanoLC 425 system (Sciex) and fractionated on a 120 mm C18 column (150 µm id column packed with 1.9 µm Reprosil-Pur 120 C18-AQ beads). Acetonitrile concentration in the mobile phase in 0.1% formic acid was increased from 5 to 18% in 88 min, to 25% at 98 min, 40% at 108 min, and to 90% at 110 min. The flow rate was 400nL/min. Peptides were electrosprayed into an SCIEX TripleTOF® 5600 mass spectrometer using an ion spray voltage of 2.5 kV, curtain gas at 35 p.s.i., nebulizer gas at 15 p.s.i., and an interface heater temperature of 150 °C. The MS survey scan range was m/z 350–1250 acquired for 250 ms. The top 20 precursor ions were selected for 85 ms per MS/MS acquisition, with a threshold of 90 counts. Dynamic exclusion was 16 s. Rolling CID function was activated, with an energy spread of 15 eV. Analysis of one sample (tangle bearing neurons) failed due to technical problems and was removed from the analysis leaving an *n* = 11 for the tangle bearing neurons.

### Protein inference and relative protein quantification

Raw files were first converted from WIFF to mzML using ProteoWizard's qtofpeakpicker (version 3) with parameters" –resolution 20,000–area –threshold 1–smoothwidth 1.1" analogous to Schubert et al. [60].

MetaMorpheus (version 0.0.311) was used to analyse the raw data [63]. The Uniprot human reference proteome database (SwissProt + TrEMBL, version 2020–04) was used to annotate spectra. Mass calibration was based on an initial search with 25 ppm mass tolerance for both precursor and product ions, the dissociation type was set to CID and Carbaminomethyl(C) and Oxidation(M) were set as fixed and variable modifications respectively. For the main search, mass tolerances from the calibration task were used and acetylation of protein N-terminus was added as a variable modification. Label-free quantification by FlashLFQ was performed at 5 ppm peakfinding tolerance with match-between-runs enabled. Normalization was disabled in MetaMorpheus. All other settings were left at default. The false discovery rate (FDR) cutoff for peptide and protein identification using MetaMorpheus was set to 1%.

### Statistical analysis of differential protein expression

MS-DAP 0.2.6.3 (https://github.com/ftwkoopmans/msdap) was used for downstream analysis of the MetaMorpheus output. In each statistical contrast of condition *A versus B*, only peptides observed in both sample group *A* and *B* in at least 2 samples were selected (per cohort). Normalization of peptide abundance values was then applied to this data subset and finally MSqRob was used for differential testing at the peptide level, accounting for the cohorts as a random variable in the regression model [13, 14]. P-values were adjusted for multiple testing with the Benjamini–Hochberg False Discovery Rate (FDR) procedure, the threshold for significance was set to 5% FDR. All statistical results are available in Table S3. The mass spectrometry proteomics data have been deposited to the ProteomeXchange Consortium via the PRIDE [51] partner repository with the dataset identifier PXD023199.

### Immunohistochemical analysis

Paraffin embedded human hippocampus, of 71 cases representing all Braak stages for NFT pathology, was cut (5 µm) and the sections were placed on SuperFrost microscope slides (VWR) and air-dried overnight at 37 °C. Prior to staining the paraffin was removed by washing in xylene. Next, the sections were washed in decreasing concentrations of ethanol (100, 96 and 70% (v/v)). Endogenous peroxidase activity was quenched by incubating in methanol with 0.3% H_2_O_2_ for 30 min at RT. Next, antigen retrieval was performed by submerging the slides in citrate buffer (pH 6) and heating in an autoclave to 126 °C.

Primary antibodies (Table 2, Table S4) were diluted in normal antibody diluent (ImmunoLogic, Duiven, The Netherlands) and incubation was performed overnight at RT. After incubation the sections were thoroughly washed in PBS (pH 7.4), 3 times for 10 min followed by incubation with an HRP-labelled secondary antibody (Envision, DAKO) for 30 min. Again, the sections were thoroughly washed in PBS (pH 7.4) 3 times for 10 min and then incubated with DAB to visualize antibody binding. Counterstaining of the nuclei was performed by incubation in hematoxylin for 3 min followed by extensive washing in running tab water for 5 min. Next, the sections were dehydrated by incubation in increasing concentrations of ethanol consisting of 70% (v/v), 96% (v/v) and 100% (v/v) ethanol. The slides were then incubated in xylene and mounted using Quick-D mounting medium. A negative control was made by omission of the primary antibody. Primary antibodies used for data presented in the figures are present in Table 2 and all tested antibodies are listed in Table S4. Optimization of antibody dilution was achieved by titration of antibodies using both control and AD brain tissue.

**Table 2:** Antibodies used in this study

Gene	Manufacturer	Order nr	Clone	Species	Dilution IHC	Immunoblot	Fast IHC for MS
CK1δ	Santa Cruz	sc-55553	C-8	Mouse	1:800		1:25
Ck1ε	Santa Cruz	sc-25423	Polyclonal	Rabbit		1:1000	
pTau	ThermoFisher	MN1020	AT8	Mouse	1:800		1:100
VXN	OriGene	TA334828	Polyclonal	Rabbit	1:800	1:1000	
PPIA	Abcam	ab42408	Polyclonal	Rabbit	1:800		
TOMM34	Santa Cruz	sc-101284	S-05	Mouse	1:6400		
TOMM34	Protein Tech	12196–1-AP	Polyclonal	Rabbit	1:6400		
HSP70	Santa Cruz	sc-24	W27	Mouse	1:200	1:1000	
CHMP1A	Santa Cruz	sc-271617	B-5	Mouse	1:12800		
TPPP	Santa Cruz	sc-515819	A-6	Mouse	1:3200		
CNDP2	Protein Tech	14925–1-AP	Polyclonal	Rabbit	1:6400		
PPIB	R&D systems	MAB5410	549205	Mouse		1:1000	

### Assessment of the percentage of neurons with immunoreactivity for pTau and markers for GVD bodies

The total number of neurons was determined in the CA1 and subiculum regions of the hippocampus. Unstained neurons were identified based on their morphology and the size of the nucleus. The number of neurons containing pTau (AT8) immunoreactivity or typical granular immunostaining of CK1δ, CK1ε, TOMM34 and VXN and a visible nucleus were determined. Differences between groups were identified using an ANOVA followed by Bonferroni’s test for multiple comparisons. A p-value of < 0.05 was considered significant.

### Immunoblotting analysis

Protein extracts were prepared by lysis of hippocampal tissue in reducing SDS sample buffer. Proteins were denatured at 95 °C for 5 min separated by SDS-PAGE using Criterion™ TGX stain-free™ precast gels (Bio-Rad, Hercules, CA, USA) and transferred (100 V for 2 h at 4 °C) onto a 0.45 µm PVDF membrane (Merck Millipore), which was pre-incubated in 100% methanol. Blocking of the membrane after transfer, was achieved using Odyssey blocking buffer (LI-COR). Subsequently the membrane was incubated with the primary antibody overnight. Primary antibodies and dilutions are listed in Table 2.

After washing in Tris-buffered saline (pH 7.5) with 0.1% (v/v) Tween-20 (TBST) for 3 × 10 min, the membrane was incubated for 3 h with the secondary antibody. Secondary antibodies used were IRDye 800 CW Goat anti-Rabbit (LI-COR Biosciences, Lincoln, NE, USA) and IRDye 680 Goat anti-Mouse (LI-COR Biosciences, Lincoln, NE) both at a 1:7000 dilution in Odyssey blocking buffer. Visualization was achieved using an Odyssey imaging system using excitation wavelengths of 700 and 800 nm. Total protein load was visualized using a chemidoc EZ (Bio-Rad) and the protein densitometric values were then used to normalize for their relative protein input. Quantification was performed using image-J software.

### Gene ontology and pathway analysis

Gene ontology and pathway analysis was performed using g-profiler [55, 56]. The following databases were included: Gene ontology (GO), molecular function (MF), GO cellular component (CC), GO biological process (BP), KEGG pathways and Reactome pathways. G-profiler databases were updated July 2020. All detected proteins were used as a background dataset. Multiple testing corrected p-value was determined using Benjamini Hochberg FDR where an adjusted *p*-value of < 0.05 was considered significant.

## Results

The proteomes of three populations of neurons from human post-mortem hippocampal tissue were assessed. These were (I) neurons with GVD marked by CK1δ positive granules (*n* = 12) and (II) pTau (AT8) positive tangle bearing neurons, both from AD cases (*n* = 12), and (III) neurons isolated from cognitively healthy control cases that contained no, or very little, AD related pathology (*n* = 12) (Fig. 1). This analysis was performed in two separate batches containing an *n* = 6 per group. Per case and for each sample 3000 neurons were analysed, resulting in an average quantification of 1981 (SD = 137.5) proteins in control, 2034 (SD = 120.9) proteins in CK1δ positive and 1935 (SD = 146.6) proteins in pTau positive neurons in batch 1 and 2185 (SD = 62.8) proteins in control, 2177 (SD = 46.5) proteins in CK1δ positive and 2150 (SD = 24.1) proteins in pTau positive neurons in batch 2. The integrated analysis accumulated in detecting a total of 2596 different proteins. There was no significant difference between the groups in the number of proteins identified within each batch (Fig. S1).

### Proteins with altered abundance

**Fig. 2:**
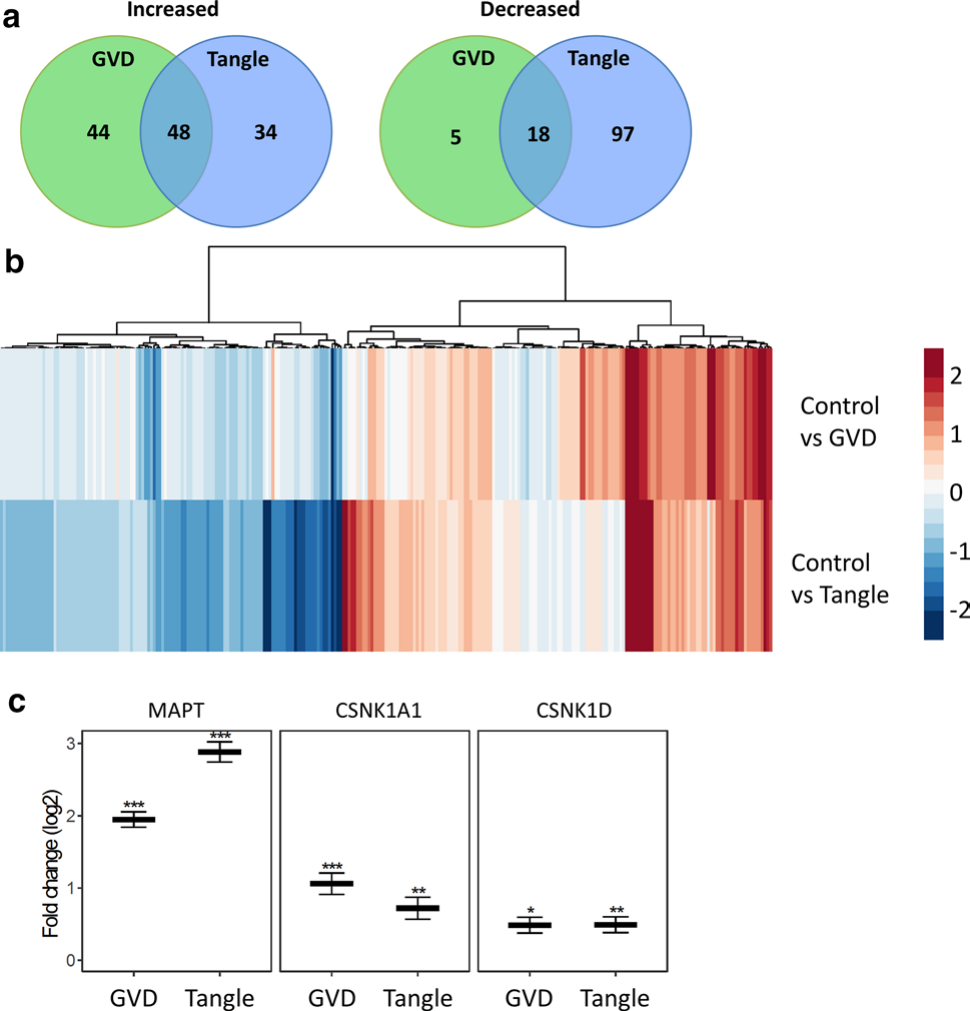
Data overview. Shown are the number of proteins increased and decreased in each population and its overlap (**a**). The protein expression profile based on fold changes in GVD and tangle bearing neurons compared to control. Most differentially expressed proteins change in the same direction in GVD and tangle bearing neurons, in which tangle bearing neurons generally exhibit more severe changes. Few proteins are increased predominantly in GVD (**b**). Log2 fold changes of known markers of GVD, CSNK1A1 (CK1α) and CSNK1D (CK1δ) and tangles, MAPT (tau) are shown (**c**)

To identify differentially expressed proteins in neurons with GVD or tangle bearing neurons compared to control a regular statistical approach was used for proteins with quantitative data in at least two cases per group (Fig. 1b). We identified 92 proteins with significant higher expression level and 23 proteins with lower levels in the GVD bearing neurons and 82 proteins with significant higher expression level and 115 proteins with lower levels in the tangle bearing neurons, compared to control neurons (Fig. 2a). Proteins with increased expression in GVD compared to control are shown in Table 3. All proteins with an altered abundance in GVD and/or tangle bearing neurons are listed in Table S2, the complete dataset, with log2 fold changes and q-values in Table S3.

**Table 3:** Proteins increased in GVD

Gene	Log2 FC C vs GVD	Log2 FC C vs Tangle	FDR C vs GVD	FDR C vs Tangle
MAPT	1.949	2.885	0.000	0.000
BRD2	1.785	0.404	0.004	ns
ARHGDIA	1.660	1.463	0.000	0.001
UBE2QL1	1.553	0.932	0.000	0.012
GFAP	1.538	1.575	0.000	0.001
PPIA	1.537	1.344	0.001	0.003
UBB*	1.434	2.051	0.000	0.000
CHMP1A	1.358	0.744	0.000	0.034
UNC13D	1.297	1.477	0.043	0.014
FKBP2	1.188	0.490	0.003	ns
PPIB	1.142	0.768	0.000	0.010
ENO1	1.128	1.070	0.012	0.026
PHPT1	1.065	0.000	0.005	ns
CSNK1A1	1.060	0.721	0.000	0.001
TOLLIP	1.006	0.933	0.023	0.028
PPIA	0.981	0.769	0.013	0.044
PEA15	0.980	0.838	0.000	0.000
PGAM1	0.957	0.914	0.000	0.001
TOMM34	0.944	0.793	0.000	0.000
CALR	0.928	0.416	0.002	ns
ENSG00000276612	0.862	0.797	0.003	0.045
HSPB1	0.807	1.064	0.000	0.000
CNRIP1	0.799	0.693	0.008	ns
PSD3	0.777	0.510	0.000	0.032
PITHD1	0.776	0.000	0.023	ns
ENO2	0.764	0.700	0.008	0.013
CLU	0.763	0.820	0.000	0.000
CBR1	0.755	0.637	0.000	0.009
HPCAL4	0.753	0.624	0.007	0.033
ENO1	0.752	0.829	0.015	0.005
MAP2	0.709	0.441	0.000	0.014
HPCA	0.709	0.432	0.019	ns
PAFAH1B3	0.709	0.365	0.001	ns
MLF2	0.704	0.409	0.000	0.040
DNAJB6	0.666	0.472	0.000	0.005
VIM	0.646	0.832	0.000	0.000
G3BP2	0.644	0.213	0.005	ns
SCRN1	0.644	0.530	0.005	0.034
CNDP2	0.642	0.642	0.003	0.016
SNX3	0.636	0.302	0.000	ns
EIF4H	0.608	0.439	0.000	0.007
GANAB	0.606	0.181	0.005	ns
HSD17B10	0.598	0.201	0.018	ns
AIMP1	0.577	0.000	0.020	ns
YWHAZ	0.569	0.498	0.002	0.006
CKB	0.568	0.391	0.044	ns
TKT	0.563	0.459	0.005	0.037
HSPA1B	0.554	0.445	0.003	0.020
EEF1B2	0.553	0.000	0.003	ns
TPI1	0.538	0.276	0.034	ns
PRDX5	0.533	0.468	0.008	0.033
GSN	0.523	0.437	0.005	0.064
VTI1B	0.519	0.161	0.044	ns
PALM	0.515	0.825	0.029	0.001
KARS1	0.514	0.183	0.001	ns
BLVRB	0.511	0.629	0.000	0.000
NEFL	0.502	0.398	0.000	0.015
CSNK1D	0.488	0.492	0.001	0.001
PAFAH1B1	0.478	0.325	0.044	ns
NEFM	0.478	0.362	0.000	0.059
SSB	0.478	0.000	0.001	ns
TPPP	0.473	0.462	0.022	0.008
PDIA3	0.456	0.000	0.004	ns
YWHAE	0.449	0.384	0.000	0.001
FSCN1	0.447	0.545	0.026	0.005
SYN1	0.441	0.458	0.043	0.062
PRKRA	0.438	0.000	0.004	ns
RTRAF	0.429	0.194	0.042	ns
AK1	0.405	0.368	0.003	0.020
NCL	0.398	0.000	0.013	ns
SNX12	0.392	0.259	0.026	ns
SDCBP	0.388	0.193	0.027	ns
EIF3CL	0.385	0.000	0.044	ns
CLINT1	0.379	0.000	0.017	ns
ANXA5	0.374	0.378	0.036	ns
INA	0.370	0.288	0.004	ns
CFL1	0.366	0.000	0.019	ns
PIN1	0.362	0.261	0.004	ns
MAP1B	0.352	0.072	0.002	ns
ATP6V1E1	0.341	0.400	0.008	0.022
VTA1	0.322	0.225	0.026	ns
CRYAB	0.321	0.576	0.048	0.043
HSPA8	0.295	0.331	0.046	0.006
ME1	0.290	0.071	0.001	ns
CAMK2D	0.289	0.070	0.026	ns
SND1	0.281	0.000	0.004	ns
LRRC47	0.275	0.000	0.005	ns
SUGT1	0.251	0.216	0.037	0.043
USO1	0.238	0.000	0.005	ns
PCBP1	0.222	0.000	0.020	ns
RAB6A	0.208	0.267	0.019	0.033
RTN4	0.207	0.129	0.044	ns

All proteins that have an increased abundance in GVD are shown. The full set of regulated proteins is presented in Table S2. *Indicates this is the leading “razor protein”, but based on the peptide data these proteins cannot be distinguished from some other highly similar proteins. These are presented in Table S3. *FC* fold change, *FDR* false discovery rate, *ns* not significant, *GVD* granulovacuolar degeneration, *C* control

Within the differentially expressed groups of proteins, GVD and/or tangle bearing neurons versus controls, there are proteins unique to each group and proteins that are shared in GVD and tangle bearing neurons (Fig. 2a). There are relatively few proteins with decreased expression in the GVD positive neurons whereas in the tangle bearing neurons many proteins with decreased expression are observed.

The protein expression profile based on fold changes in GVD and tangle bearing neurons compared to control shows that most changes that occur in GVD and tangle bearing neurons are in the same direction, where the tangle bearing neurons generally show the largest change versus control. Only a small number of proteins is increased in GVD but not or less increased in tangle bearing neurons (Fig. 2b).

### Higher abundance and differential localization for selected proteins

Differentially expressed proteins included those known to be increased in AD, for example CSNK1A1 (CK1α), CSNK1D (CK1δ) and MAPT (Tau), which are associated with GVD and tau pathology respectively (Fig. 2d). The abundance of CK1δ and CK1α is increased in GVD bearing neurons and for CK1α to a lesser extent in tangle bearing neurons. MAPT on the other hand is increased in GVD and even more so in tangle bearing neurons.

**Fig. 3:**
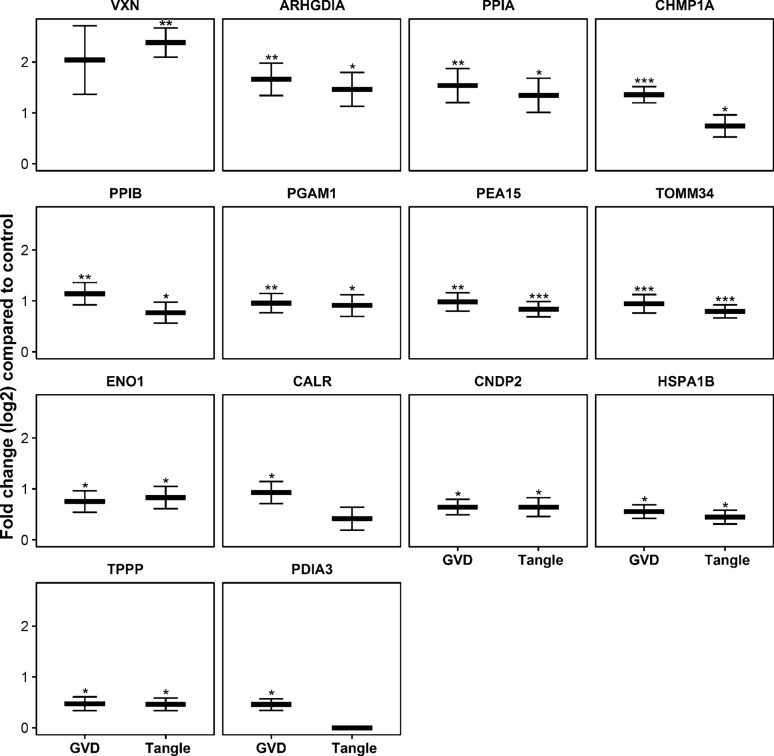
Proteins selected for immunohistochemical analysis. Thirteen proteins that were found increased in neurons with GVD using the statistical approach, were selected for further analysis using immunohistochemistry (IHC). VXN was included for its high estimated fold change and a large increase in the number of individual peptides that was detected in GVD compared to control, indicated by a high z-score, suggestive of an increase in GVD. The log2 fold changes are indicated in GVD and tangles compared to control (a). *q < 0.05, **q < 0.001 and ***q < 0.0001

**Fig. 4:**
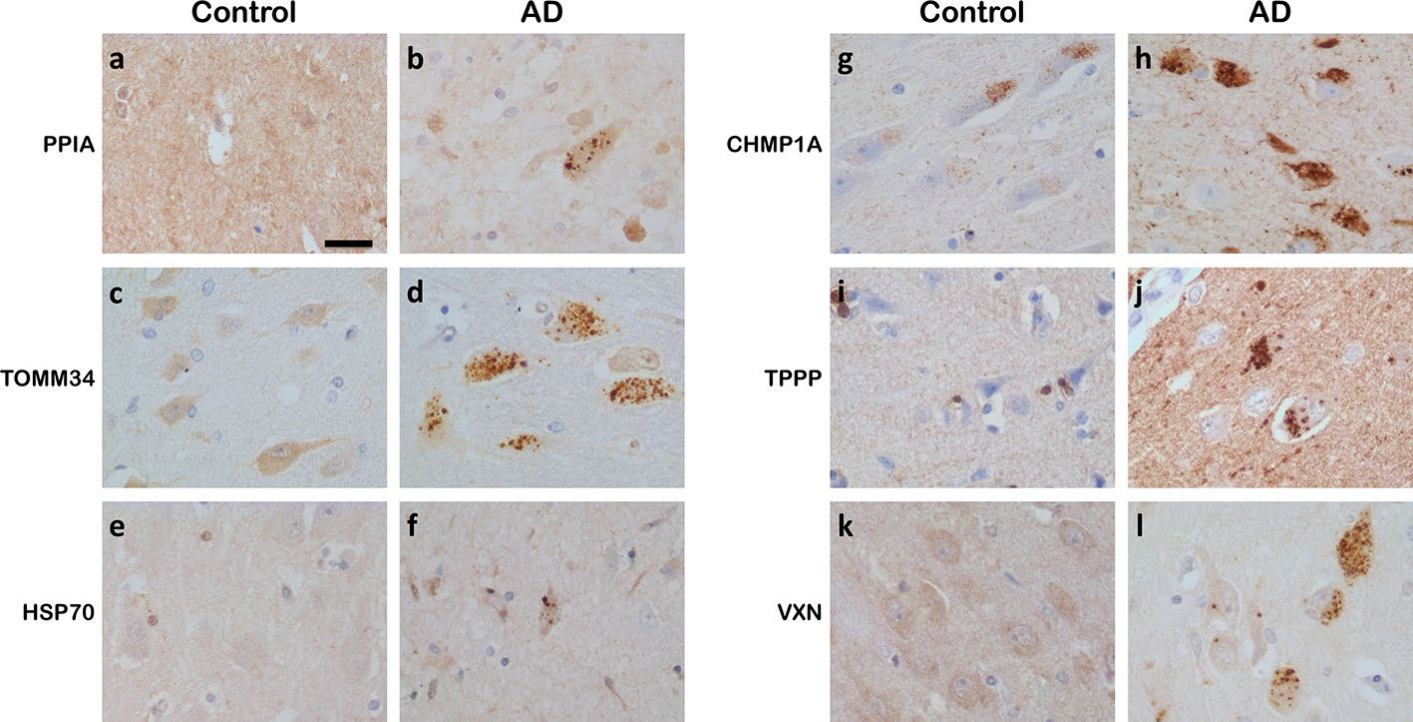
Validation of differentially expressed proteins in GVD bearing neurons. Representative images are shown from immunohistochemical staining for proteins that are differentially expressed (PPIA, TOMM34, HSP70, CHMP1A, TPPP and VXN) in control cases (**a**, **c**, **e**, **g**, **I**, **k** and **m**, respectively) and AD cases (**b**, **d**, **f**, **h**, **j**, **l** and **n**, respectively). PPIA, TOMM34, HSP70, CHMP1A, TPPP, and VXN are localized in the GVD bodies in AD. All images are taken in the CA1 or subiculum subregion of the hippocampus. Scale bar in (**a**) indicates 25 μm

Based on an increase in protein level in neurons with GVD and on antibody availability, 13 proteins were assessed by IHC (Figs. 3 and 4). Protein association with GVD and/or AD was confirmed by IHC with antibodies (Table 1) for PPIA, TOMM34, HSP70 (HSPA1B), CHMP1A and TPPP in at least *n* = 3 per group. All tested antibodies are listed in Table S4. In addition, for proteins that have very low abundance in a particular group, proper statistical testing can be challenging as only few data points are available for statistics. In that case, differences in the number of peptides that are detected can be a useful indicator of a potential change in expression. This differential detection in the number of individual peptides is then indicated as a z-score (Table S3). An example of a protein with a high z-score and data suggestive of a high increase in GVD is VXN (a.k.a. C8orf46), which was confirmed by additional IHC staining.

PPIA shows a strong increase in neuronal immunoreactivity that is localized to the GVD bodies (Fig. 4a, b). TOMM34 shows weak diffuse immunoreactivity across the neuronal cell body in control neurons. In AD, very strong immunoreactivity is present in cells with GVD bodies and the anti-TOMM34 antibody specifically stains GVD structures (Fig. 4c, d). Similar staining was observed using two different TOMM34 antibodies (Fig S2). HSP70 (HSPA1B) shows very little immunoreactivity in control cases and is increased in AD. Localization is mostly related to neurons, staining a proportion of the GVD bodies, and some glial cells (Fig. 4e, f). GVD is also visualized using 3 other HSP70 antibodies tested in this study (Fig S3). CHMP1A shows a granular staining pattern in control neurons. Surrounding the neurons also some immunoreactivity is found, that is possibly related to cellular protrusions. In AD, the level of immunoreactivity is largely increased in neurons, and mainly associated with cells containing GVD bodies and to a large extent localized in the GVD bodies (Fig. 4g, h). TPPP immunoreactivity is present in GVD bodies and in non-neuronal cells (Fig. 4i, j), presumably oligodendrocytes as the staining pattern matches a previous study by Kovacs et al. [32]. For VXN we observed a weak diffuse primarily neuronal staining in control tissue. An increase in immunoreactivity is seen in AD, that appears specifically localized in GVD bodies (Fig. 4m, n). In summary, IHC analysis of selected proteins that were found increased in laser-dissected GVD containing neurons, shows higher intensities in AD cases compared to control case and confirms the presence of these proteins in GVD.

When performing quantification of CK1ε, VXN, TOMM34 and PPIB using immunoblotting on whole hippocampal lysates of 7 control cases versus 6 AD cases, no significant change in abundance was found (Fig S4), indicating that immunoblotting of whole hippocampal lysates does not provide sufficient resolution to match the laser-dissected cellular proteomics approach.

### VXN and TOMM34 increase with pathology and parallel the presence of CK1δ, CK1ε and pTau in the hippocampus

**Fig. 5:**
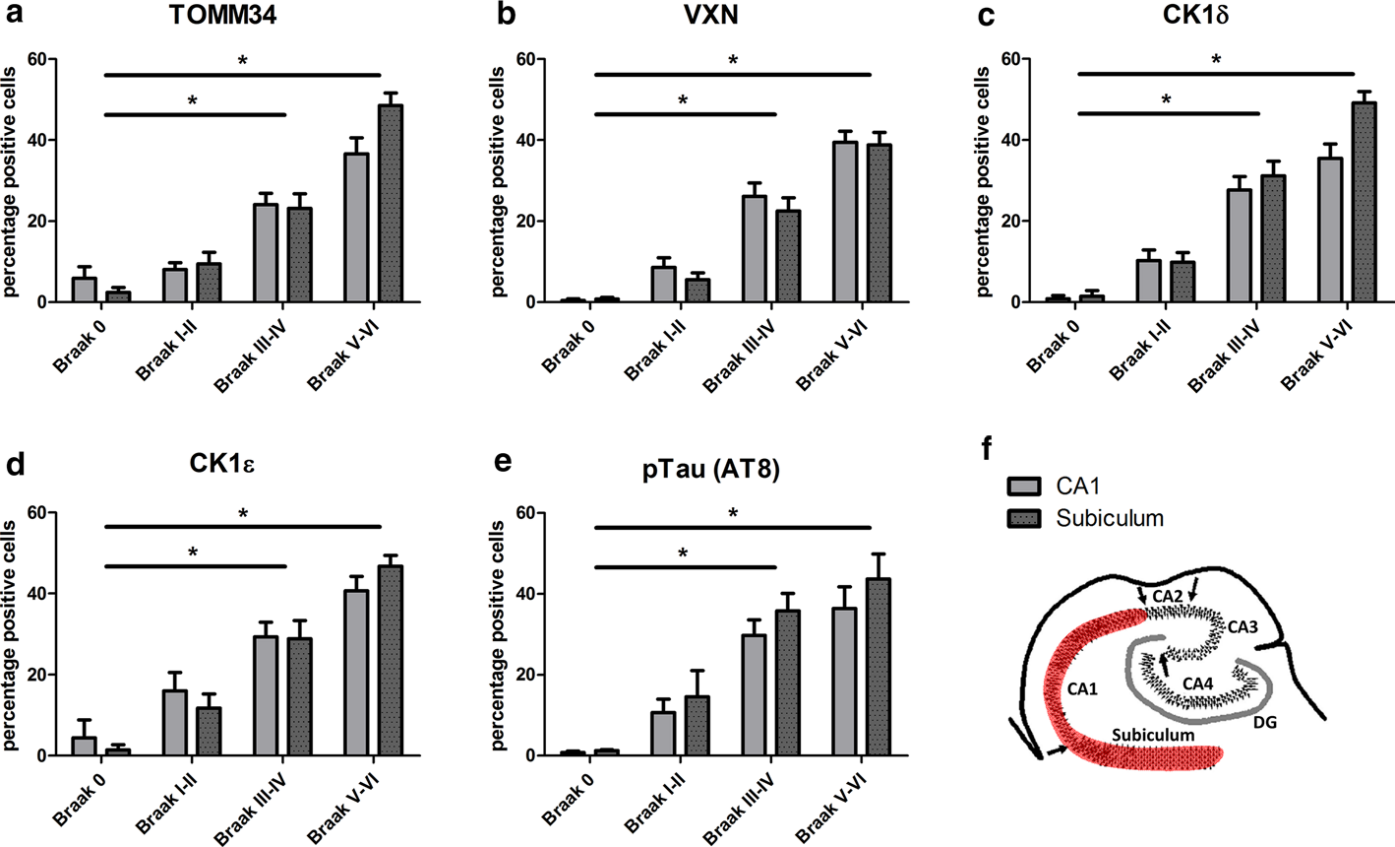
Percentage of neurons containing pTau and markers for GVD bodies over the Braak stages. In the hippocampal subregions CA1 and Subiculum the percentage of neurons that displayed the typical GVD associated granular staining pattern was determined for new markers for GVD (TOMM34 and VXN) (**a**, **b**) and known markers of GVD (CK1δ, CK1ε) (**c**, **d**). In addition, the percentage of neurons that are positive for pTau (AT8) (**e**) was determined. A schematic representation of a human hippocampus with the quantified area marked in red is shown in (**f**). Significant differences (*p* < 0.05) compared to Braak stage 0 are indicated with an asterisk

Next, we selected the newly discovered markers VXN and TOMM34, which showed a high degree of association with GVD, to stain a large patient cohort (*n* = 71), encompassing all Braak stages for NFT to quantify the percentage of positive neurons. The percentage of stained neurons was determined in the CA1 and subiculum subregion of the hippocampus (Fig. 5). Over the Braak stages there is a progressive increase in the fraction of neurons that are positive for TOMM34 and VXN. This increase proceeds to affect approximately 50% of all neurons in the hippocampus. The rate and extent of increase in percentage positive cells was similar to that of the known GVD associated proteins CK1δ and CK1ε and pTau (AT8) (Fig. 5).

### Processes associated with GVD and/or tangle bearing neurons

To obtain insight in disease mechanisms occurring in GVD and tangle bearing neurons an overrepresentation analysis in G-profiler was performed on significantly regulated proteins (Table S2). Separate subgroups defined as (I) increased in GVD, (II) decreased in GVD, (III) increased in tangle bearing neurons and (IV) decreased in tangle bearing neurons were analysed (Table S5).

(I) Considering all 92 proteins that are increased in GVD, this yielded 52 overrepresented terms with intermediate filament-based process being the most significant biological process. (II) Proteins that are decreased in GVD containing neurons provided no enriched terms in our analysis. (III) Proteins increased in tangle bearing neurons yielded 159 GO and pathway terms, with neurogenesis being the most significantly affected biological process. (IV) Proteins that were decreased in tangle bearing neurons primarily showed an overrepresentation in 251 terms with RNA catabolic process being the most significant biological process, but also included ribosomal proteins and cellular responses to stress.

Combining increased and decreased proteins in GVD or increased and decreased proteins in tangle bearing neurons did not result in any additional overrepresented GO terms or pathways for GVD but 49 additional significant terms for tangle bearing neurons were found (Table S5).

**Fig. 6:**
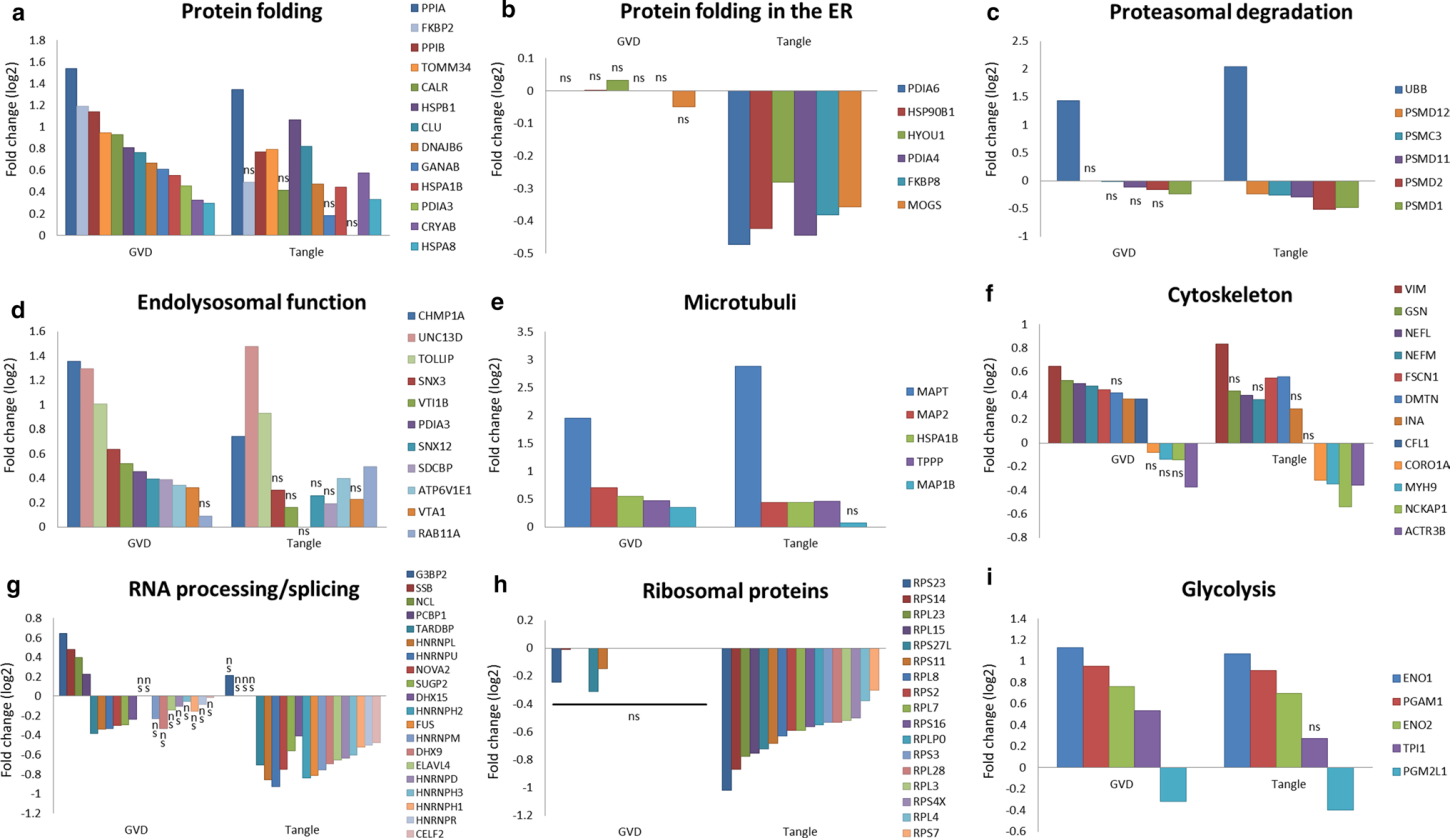
Fold changes of selected proteins that belong to specific functional groups. Log2 fold changes compared to control are shown for proteins of different functional categories in (**a**–**i**). We detected increased levels of proteins related to protein folding in neurons with GVD compared to control while levels are generally lower in tangles compared to GVD (**a**). Several other proteins involved in protein folding, which in this case all function in the endoplasmic reticulum, remain unchanged in neurons with GVD but are reduced in tangles (**b**). Core components of the proteasome are found to be decreased in GVD and tangles while ubiquitin (UBB) builds up in GVD and to a greater extent in tangles (**c**). Several proteins related to the endolysosomal pathway are changed in expression, primarily in GVD and to a lesser extent in tangle bearing neurons (**d**). Several proteins related to the microtubule **e** and other neuronal cytoskeletal components **f** are elevated in GVD and tangles. Many proteins that are involved in RNA processing including several members of the Heterogeneous nuclear ribonucleoproteins (HNRNPs) are decreased in neurons with GVD and tangles, except for Nucleolin (NCL), Ras GTPase-activating protein-binding protein 2 (G3BP2), Lupus La protein (SSB) and heterogeneous nuclear ribonucleoprotein E1 (PCBP1) which are increased in GVD but unchanged in tangles (**g**). Several ribosomal proteins are decreased but only in tangles (**h**). Several enzymes facilitating sequential steps in glycolysis namely Alpha-enolase (ENO1), Phosphoglycerate mutase 1 (PGAM1), gamma-enolase (ENO2) and triosephosphate isomerase (TPI1) are increased in GVD and all, except for TPI1, also in tangles, while glucose 1,6-bisphosphate synthase (PGM2L1) was found decreased in GVD and tangle bearing neurons (**i**). (*ns* not significant, *nd* not detected)

In addition to the overrepresentation analysis highlighting the proteins affected in GVD and tangles, an extensive, neurodegeneration relevant, overview and visualization of functionally related proteins and their changes in abundance related to GVD and tangles is presented (Fig. 6a–i). These include proteins involved in protein folding, proteasomal degradation, RNA processing, ribosome function, microtubule and cytoskeletal-related functions, endo-lysosomal function and glycolysis.

In AD, and neurons with GVD, accumulation of misfolded proteins occurs [19, 77]. An important response to misfolded proteins is via proteins that are involved in protein folding, as supported by GO/pathway analysis. Such proteins include PPIA, PPIB, CALR, HSPB1, CLU, DNAJB6, GANAB, HSPA1B, PDIA3, CRYAB, HSPA8 (GO:0006457), FKBP2 [78] and TOMM34 [9]. The mean fold change of each protein per group shows an increase in neurons with GVD (Fig. 6a), whereas the increase in tangle bearing neurons is often less or not significant, appearing as an up-down profile when assuming consecutive stages from GVD to tangle bearing neurons. The ER resident proteins PDIA6, HSP90B1, HYOU1, PDIA4, MOGS and FKBP8, have functions involving protein folding and the unfolded protein response. These proteins are decreased only in tangle bearing neurons (Fig. 6b, Table S2). A well-known mechanism of removal of misfolded proteins is by proteasomal degradation. We observe a change in the abundance of proteins that are components of the proteasome complex including PSMC3, PSMD1, PSMD2, PSMD11, and PSMD12 and an increase of ubiquitin (UBB), the latter indicating accumulation of ubiquitinated proteins (Fig. 6c, Table S2). Misfolded tau is also being degraded via the endolysosomal pathway and is found in early endosomes, multivesicular bodies, and lysosomes [71]. Several proteins related to this pathway (GO:0005764 lysosome, GO:0005768 endosome, GO:0005771 multivesicular body) are found regulated in neurons with GVD (Fig. 6d). These include CHMP1A, SNX3, VTI1B, PDIA3, SNX12, VTA1, RAB11A, UNC13D, TOLLIP, SDCBP and ATP6V1E1.

Regarding the neuronal cytoskeleton several proteins that are related to the microtubule, including MAPT, are found to have an altered abundance (Fig. 6e, Table S2). Also related to the actin cytoskeleton we find increased GSN, and FSCN1 and CFL1 as well as the neurofilament components VIM, NEFL, NEFM and INA in GVD and partially in tangles (Fig. 6f). A decrease in expression was found for actin cytoskeleton regulators CORO1A, MYH9 and NCKAP1 in tangles and for ACTR3B in both GVD and tangles.

The abundance RNA processing proteins, specified by presence in “mRNA binding” (GO:0003729), “RNA splicing” (GO:0008380), “RNA processing” (GO:0006396), is changed in GVD and to even greater extent in tangles (Fig. 6g).

Ribosomal proteins as assigned to “structural constituent of ribosome” (GO:0003735) include RPS2, RPS3, RPL4, RPS4X, RPS7, RPS11, RPS14, RPS16, ENSG00000260836 (40S ribosomal protein S17), RPS23, RPS27L, RPL3, RPL7, RPL8, RPL15, RPL23, RPL28 and RPLP0 which are all decreased but only in the tangle bearing neurons (Fig. 6h). This indicates that in tangle bearing neurons protein synthesis might be impaired. We observe an increase in ENO1, PGAM1, ENO2 and TPI1 and a decrease of PGM2L1 in GVD and tangles (Fig. 6i) with the exception of TPI which does not reach significance in tangle bearing neurons. These proteins are facilitating sequential steps in glycolysis, the main energy source for the brain.

Interestingly, no proteins were found that are increased in GVD and decreased in tangle bearing neurons when compared to control, indicating no contradictory responses are found when comparing GVD to tangle bearing neurons.

## Discussion

Mass spectrometry-based proteomics analysis of laser dissected tissue or cells has become a useful technology to identify proteins specifically in pathological conditions [7, 16, 20]. In this study we applied a single-cell resolution proteomics approach that provides for the first time an overview of changes in the proteome of neurons containing GVD and in those containing neurofibrillary tangles. Using our approach, we have identified 115 differently expressed proteins that show changes in GVD bearing neurons and 197 proteins in tangle bearing neurons of which 66 proteins overlap between GVD and tangle bearing neurons. The identification of proteins differentially expressed in GVD containing neurons provides insight in the molecular mechanisms associated with GVD.

### GVD is associated with activation of protein folding and degradation and dysregulation of RNA processing

As indicated by the GO/pathway overrepresentation analysis, several proteins associated with protein folding are increased, mostly in GVD containing neurons, and to a lesser extent in tangle bearing neurons (Fig. 6a). TOMM34 is a cytosolic protein that is involved in protein folding and the transport of unfolded proteins into mitochondria [5, 9, 42]. HSP70 binds misfolded monomeric tau, oligomeric tau and to a lesser extent aggregated tau and is effective in preventing further aggregation [33, 50, 53]. Interestingly, TOMM34 interacts with HSP70 as a co-chaperone [69] and both were found in GVD bodies using IHC. The highest fold increase is seen in the cyclophilin family of peptidyl-prolyl isomerases PPIA, PPIB and FKBP2. Interestingly, inhibition or deficiency of PPIA results in increased aggregation and faster disease progression in models for prion disease [1, 49]. Other, mainly ER resident, protein folding associated proteins maintain normal expression in GVD but decrease in tangle bearing neurons (Fig. 6b).

A downregulation of components of the proteasome complex was observed, which was accompanied by an increase of UBB (Fig. 6c) indicating proteasomal dysfunction. Increased UBB has previously been suggested to be relevant in an early stage of tangle formation [4, 74]. In addition, several proteins related to early endosomes, multivesicular bodies, and lysosomes are found regulated in neurons with GVD. Of these proteins PDIA3, which is upregulated in GVD, has previously been implied as a possible therapeutic target by reducing Aβ pathology and subsequent tau pathology in an AD model [22, 68]. In addition gene variants of the GVD increased protein SNX3 are associated with AD [70]. Together, these results indicate increased protein folding mechanisms and impaired protein degradation in GVD affected neurons.

We also observe cytoskeletal alterations of microtubule, actin and intermediate filament related proteins in GVD and continuing in tangle bearing neurons (Fig. 6f). Next to MAPT, MAP2 and MAP1B are increased in GVD and could compensate microtubule instability [27]. In addition, MAP1B is important for synaptic plasticity and neurite outgrowth [24, 48]. Also TPPP is increased and localizes in the GVD bodies. TPPP is involved in modulation of microtubule dynamics and stability [32, 47] and can bind and inhibit the activity of tau kinase glycogen synthase kinase 3 [52]. Previously the regulator of actin dynamics and synaptic plasticity CFL1 was linked to synaptotoxicity in AD and CFL1 positive inclusions were shown associated with plaque and tangle pathology in AD [38, 58, 59]. In addition, the neurofilament components VIM, NEFL, NEFM and INA are increased in GVD and NEFL is also increased in tangle bearing neurons.

GO/pathway overrepresentation analysis showed a decrease in an exceptionally large number of proteins that are involved in the binding and processing of mRNA in GVD and to an even greater extent in tangle bearing neurons. Impaired functioning of RNA processing can induce cryptic splicing errors and the relation with tau pathology has been indicated recently [21]. In GVD bearing neurons several heterogeneous ribonucleoproteins are reduced including HNRNPL, HNRNPU, while PCBP1 (hnRNP E1) is increased (Fig. 6g). Moreover, in tangles the abundance of additional related proteins from this family, including HNRNPH2, HNRNPM, HNRNPD, HNRNPH3, HNRNPH1 and HNRNPR is reduced. This family of proteins is involved in the processing, splicing, stability and transport of RNA [11, 54]. The level of TARDBP (TDP43) was found elevated in GVD and immunodetection of phosphorylated TARDBP (pTDP-43) has been found associated previously with GVD [38]. G3BP2 is a scaffold protein functioning in the development of stress granules [35]. Several stress granule markers are also found frequently present in GVD [29]. Also increase of SSB, PCBP1 and the known GVD localized protein NCL [38] in GVD suggests disturbances in RNA metabolism and protein synthesis [25]. Our findings suggest that pathways associated with RNA processing are affected already in the GVD stage of tau pathology.

VXN was found highly increased in neurons with GVD and localized specifically to the GVD bodies. As such VXN is a robust and interesting new marker for GVD. VXN has functions in the nucleus where it is involved in neurogenesis. Aberrant methylation of the VXN gene was found associated with development of ALS [41, 65].

In summary, we observed increased presence of proteins and activation of pathways that promote protein folding, synaptic plasticity, and neurogenesis which could indicate that GVD is a homeostatic response to a disturbed homeostasis in the early stages of tau pathology.

### Decreased ribosomal proteins and protein folding in the ER in tangle bearing neurons

In comparison with GVD neurons, tangle bearing neurons show further persistence or aggravation of changes in relation to protein folding, protein degradation, microtubule and other cytoskeletal proteins and RNA processing. In addition, several protein groups that were unaffected in GVD show changes in their abundance in tangle bearing neurons. Neurofibrillary tangle bearing neurons show a decrease in ER resident proteins which are involved in protein folding and the unfolded protein response (Fig. 6b). Also, a large collection of ribosomal proteins was selectively decreased in tangle neurons (Fig. 6h). Decreased synthesis of ribosomal components can be induced by misfolded, aggregated tau [8]. A decrease in ribosomal subunits may indicate a substantial reduction in the capacity for protein synthesis in tangle bearing neurons. Our data shows that the levels of these proteins are unchanged in GVD and proper ribosomal function might still be maintained in these neurons.

**Fig. 7:**
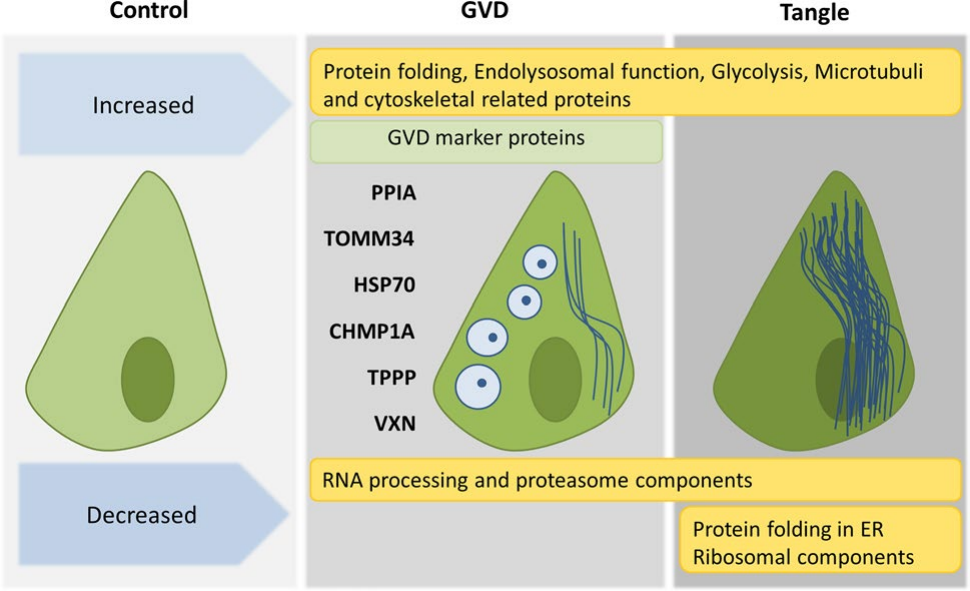
Proteins and processes associated with GVD and tangles. Shown are the global proteome changes occurring in GVD containing neurons and tangle bearing neurons. In the top panel are the processes related to proteins that are increased and at the bottom row the processes related to decreased proteins. Several markers identified using LC–MS/MS and found to be associated with GVD using IHC in this study are placed at the left of the GVD bearing neuron

There is a great deal of overlap between GVD and tangles in regulated proteins and protein groups like RNA processing, the proteasome, endolysosomal function and glycolysis as shown in Figs. 6 and 7. In addition, MAPT is the main protein aggregate in the tangle and a 3.9-fold increase is already observed in GVD bearing neurons further increasing to approximately 7.4-fold in the tangle bearing neurons. Furthermore, the aggregation of tau in tangle bearing neurons coincides with the decrease of chaperone proteins, involved in protein folding and prevention of aggregation.

Recently, Wiersma et al. showed that tau seeds induce GVD-like pathology in primary neurons in vitro [73]. This study supports a functional link between tau pathology and GVD. Although we cannot draw conclusions on the link between tau seeding and GVD in the present study, it is interesting to see that both tau seed induced GVD and GVD in human post mortem brain show strong involvement of the endolysosomal pathway. In general, both studies support the strong connection between GVD and tau pathology. These results suggest that GVD and tangle formation are sequential events.

We propose a model were tau seeds initiate the accumulation of tau, resulting in a gradual increase in tau hyper-phosphorylation and misfolding in the neuron. During this process of cellular stress, GVD is initiated. While the increase of intracellular pTau levels might elicit GVD formation, it is still elusive whether GVD is a protective or degenerative response [72].

Some limitations may apply to this work. For laser dissection of neurons with GVD or tangles, we used the markers CK1δ and pTau respectively. A proportion of neurons showing GVD are also pTau positive and vice versa, as such there will be a percentage of double positive neurons present in both groups in the analysis. In addition, proteins were solubilized using SDS buffer under reducing conditions. Although this solubilizes most proteins in a sample it does not completely solubilize protein aggregates and consequently the representation of aggregated proteins in this analysis may be incomplete. The average age of the control cases used for proteome analysis was lower than the AD cases (9.5 years difference). It was recently reported that age difference affects the brain proteome only mildly [26], and the proteins involved in that study are not part of the processes that we attribute to GVD or tangles nor do these include any of the markers highlighted in our study. Age cannot be a contributing factor in changes selective for GVD or tangle bearing neurons as these samples are derived from the same group of AD cases. However, we cannot exclude that the average age difference has an effect on the comparison between control and AD cases.

Furthermore, mass spectrometric results may require confirmation using an independent technique to assess cellular changes in protein levels. We confirmed increased abundance of several proteins not previously associated with AD or GVD by immunohistochemistry, however, changes in the expression and the localization of many other proteins of interest still need to be investigated.

Previous immunohistochemistry studies have shown the presence of phosphorylated proteins in GVD bodies. The technique used in this study was suitable for the detection of proteins independent of their phosphorylation state, which provided an unbiased analysis independent of the specificity of antibodies directed to phospho-epitopes.

For quantification by immunoblotting, whole hippocampal lysates were used as the protein yield from laser-dissected neurons is too low for this technique. The abundance of CK1ε, VXN, TOMM34 and PPIB was analysed, but no significant change in abundance was detected. This indicates that immunoblotting of whole hippocampal lysates does not provide sufficient resolution to detect changes related to the subpopulation of neurons that display GVD. This explains why we identified many proteins that have not previously been associated with AD and argues for a cell-targeted proteomics discovery approach.

For the proteins with a clear localization to the GVD bodies, like TOMM34, PPIA, VXN, or known marker proteins such as CK1d and CK1e, we do not observe significant lower abundance in tangle bearing neurons. This might be due to several reasons. First, the mass spec analysis is on a partly double positive cell population as we cannot exclude the presence of GVD in part of the isolated pTau positive neurons. Second, proteins associated with GVD might still have elevated levels of these proteins in tangle bearing neurons compared to control, but these might be below the detection threshold of the IHC analysis.

## Conclusion

Using a combination of IHC, LMD and LC–MS/MS we analysed the changes in the proteomes of neurons with GVD and neurons with tangles compared to control neurons, obtained from human post-mortem AD brain tissue. Our data show that GVD and neurofibrillary tangle bearing neurons are molecularly closely related and support that GVD represents a neuronal pre-tangle stage. Due to the high resolution of the analysis of specific neuronal populations this study identified many proteins that have not been associated previously with GVD or NFTs. Our results show that GVD bearing neurons have increased presence of proteins associated with protein folding, endolysosomal function and glycolysis, while there is a decrease of proteins involved in RNA processing and proteasome components. In addition, we find increased levels of proteins in GVD that promote protein folding, synaptic plasticity and neurogenesis suggesting that GVD is a response to a disturbed neuronal homeostasis in the early stages of tau pathology. We present new GVD associated proteins that provide insight in AD pathogenesis and novel leads for further research.

## Supplementary Information

Below is the link to the electronic supplementary material.

Supplementary file1 (DOCX 15 KB)

Supplementary file2 (TIFF 3454 KB)

Supplementary file3 (TIFF 8788 KB)

Supplementary file4 (TIFF 14690 KB)

Supplementary file5 (TIF 25632 KB)

Supplementary file6 (XLSX 13 KB)

Supplementary file7 (XLSX 43 KB)

Supplementary file8 (XLSX 370 KB)

Supplementary file9 (XLSX 12 KB)

Supplementary file10 (XLSX 1949 KB)
