# Supplementary material for: The proteome of granulovacuolar degeneration and neurofibrillary tangles in Alzheimer’s disease
Source: Acta Neuropathol. 2021 Jan 25;141(3):341–58. doi: 10.1007/s00401-020-02261-4 (PMC7882576; doi:10.1007/s00401-020-02261-4)
Supplement: Supplementary file 1 — Supplementary file1 (DOCX 15 KB) [file 401_2020_2261_MOESM1_ESM.docx]

# Text for supplementary figures

**Figure S1. Number of proteins quantified per case.** The total number of proteins and peptides that was quantified using LC/MS-MS per case is shown per individual case. Both detection by MS/MS and quantification only via the match between runs option (MS1 only) are shown (a). Samples were analysed in two separate batches with each 6 samples per group. No significant differences exist between groups within each batch with respect to number of quantified proteins (b).

**Figure S2. IHC staining using two different anti-TOMM34 antibodies visualize GVD**. Both anti-TOMM34 antibodies used in this study visualize GVD.

**Figure S3. IHC staining using 3 alternative anti-HSP70 antibodies visualize GVD.** 3 alternative anti-HSP70 andtibodies that were tested in this studie also visualize GVD (arrows).

**Figure S4. Levels in whole hippocampus are not changed as determined using immunoblotting.** Quantification of selected GVD associated proteins was performed on whole hippocampal lysates. Control (Braak 0 and I for tau pathology) was compared to AD cases (Braak stage V and VI). CK1ε, VXN, TOMM34, and PPIB were quantified and presented in a, b, c, and d, respectively. No differences in the abundance was found for any of these proteins when comparing control to AD.
