# Supplementary figures and images for: The proteome of granulovacuolar degeneration and neurofibrillary tangles in Alzheimer’s disease
Source: Acta Neuropathol. 2021 Jan 25;141(3):341–58. doi: 10.1007/s00401-020-02261-4 (PMC7882576; doi:10.1007/s00401-020-02261-4)

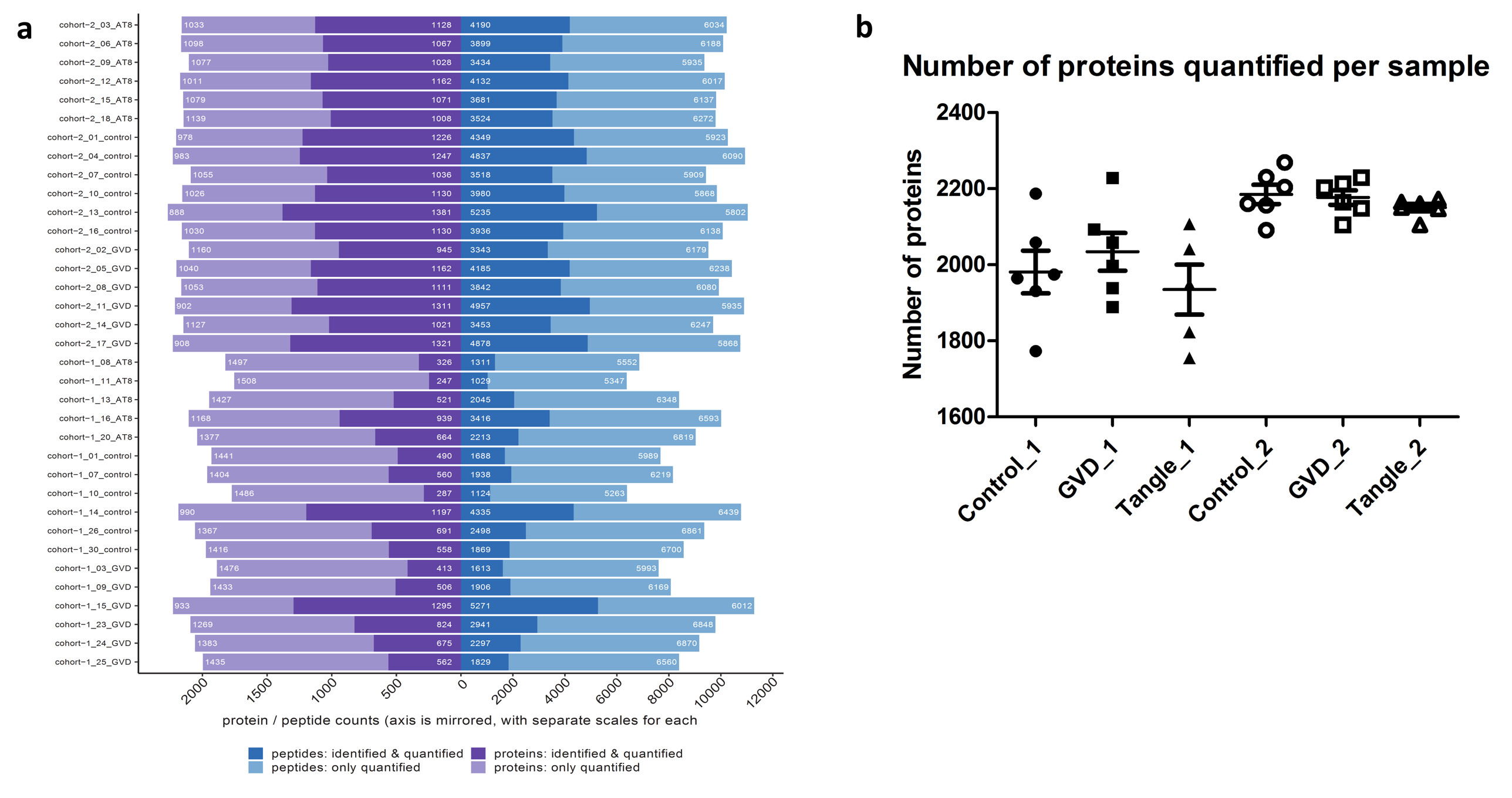

Supplement: Supplementary file 2 — Supplementary file2 (TIFF 3454 KB) [file 401_2020_2261_MOESM2_ESM.tiff]

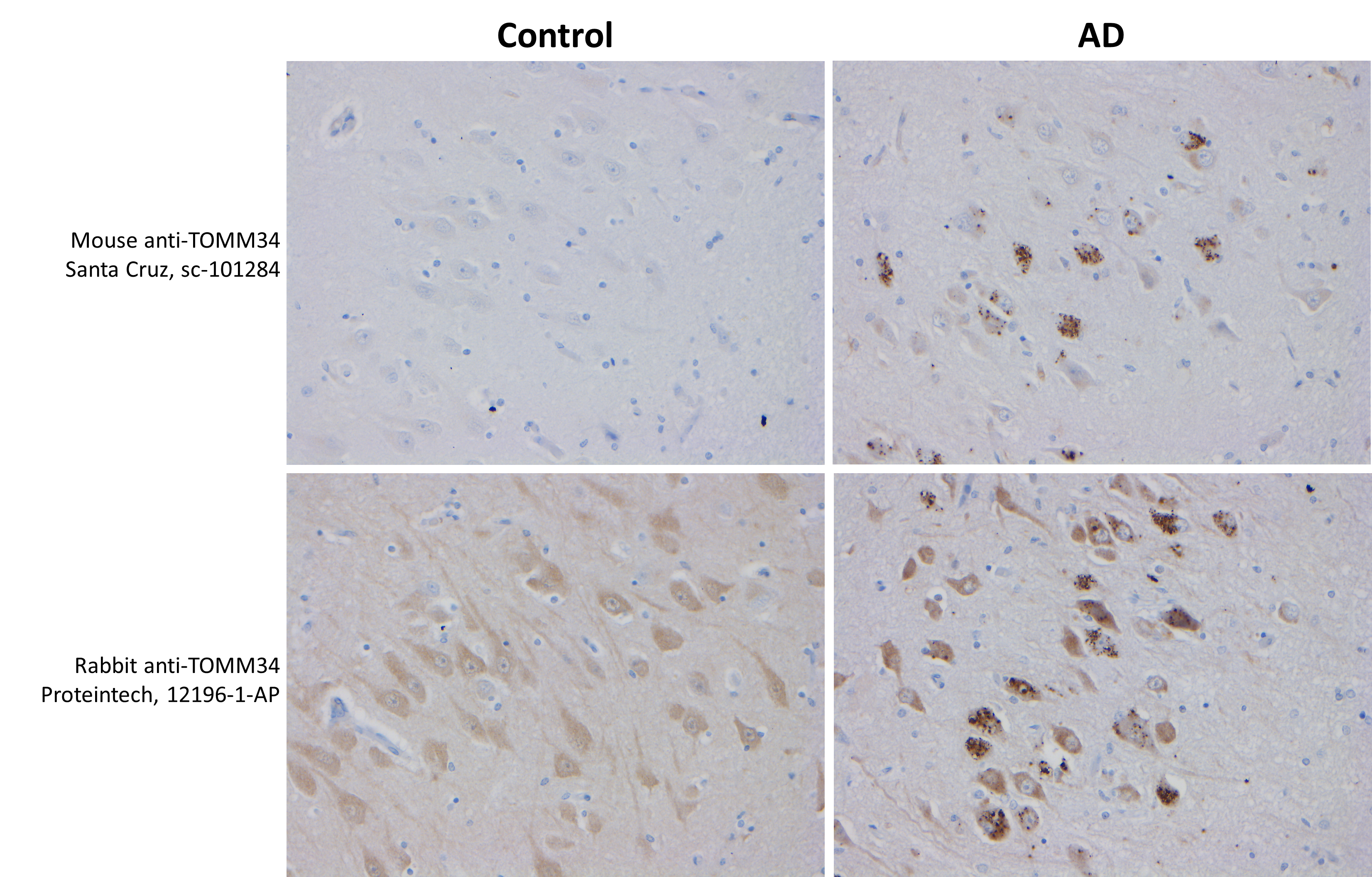

Supplement: Supplementary file 3 — Supplementary file3 (TIFF 8788 KB) [file 401_2020_2261_MOESM3_ESM.tiff]

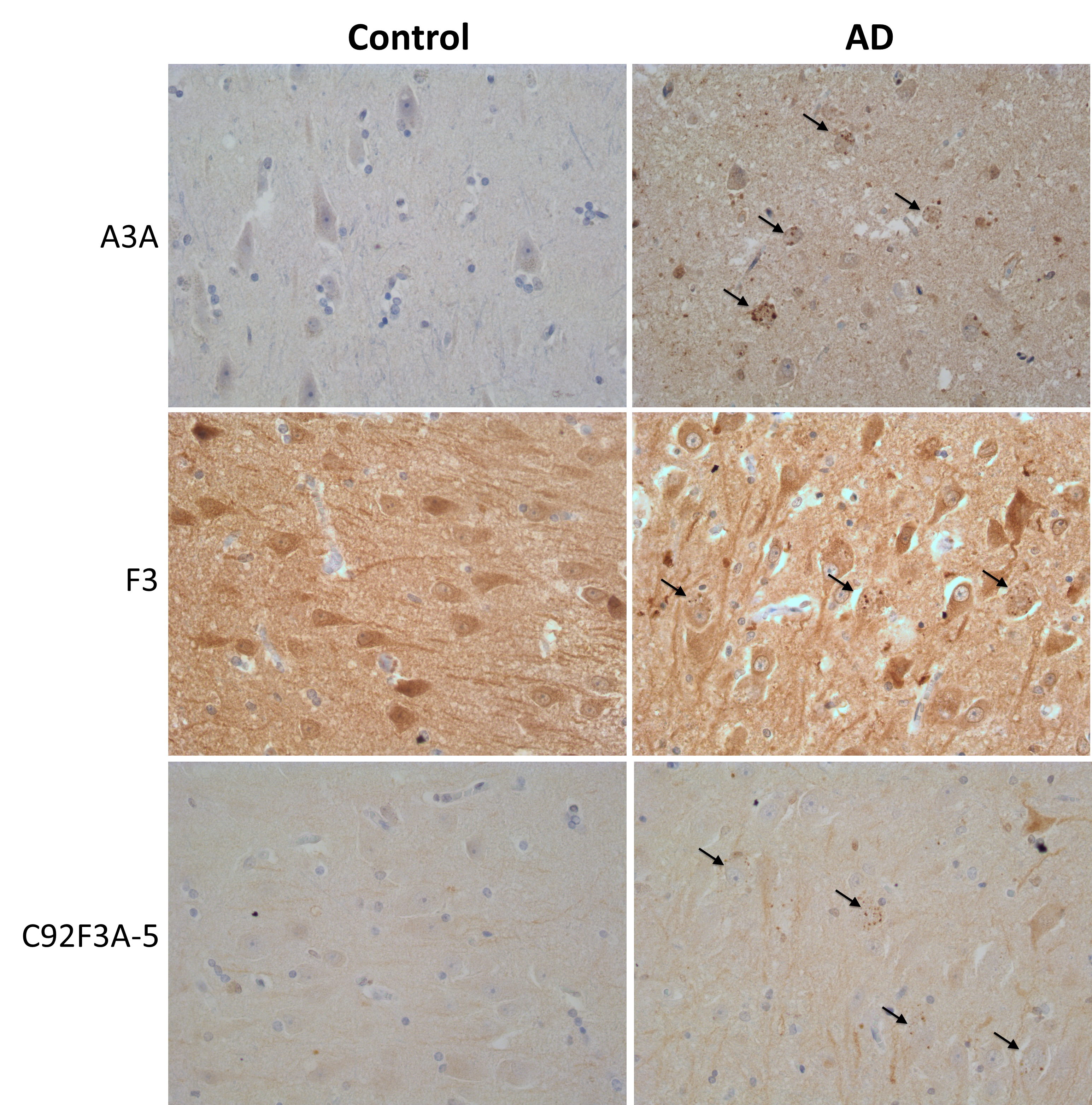

Supplement: Supplementary file 4 — Supplementary file4 (TIFF 14690 KB) [file 401_2020_2261_MOESM4_ESM.tiff]

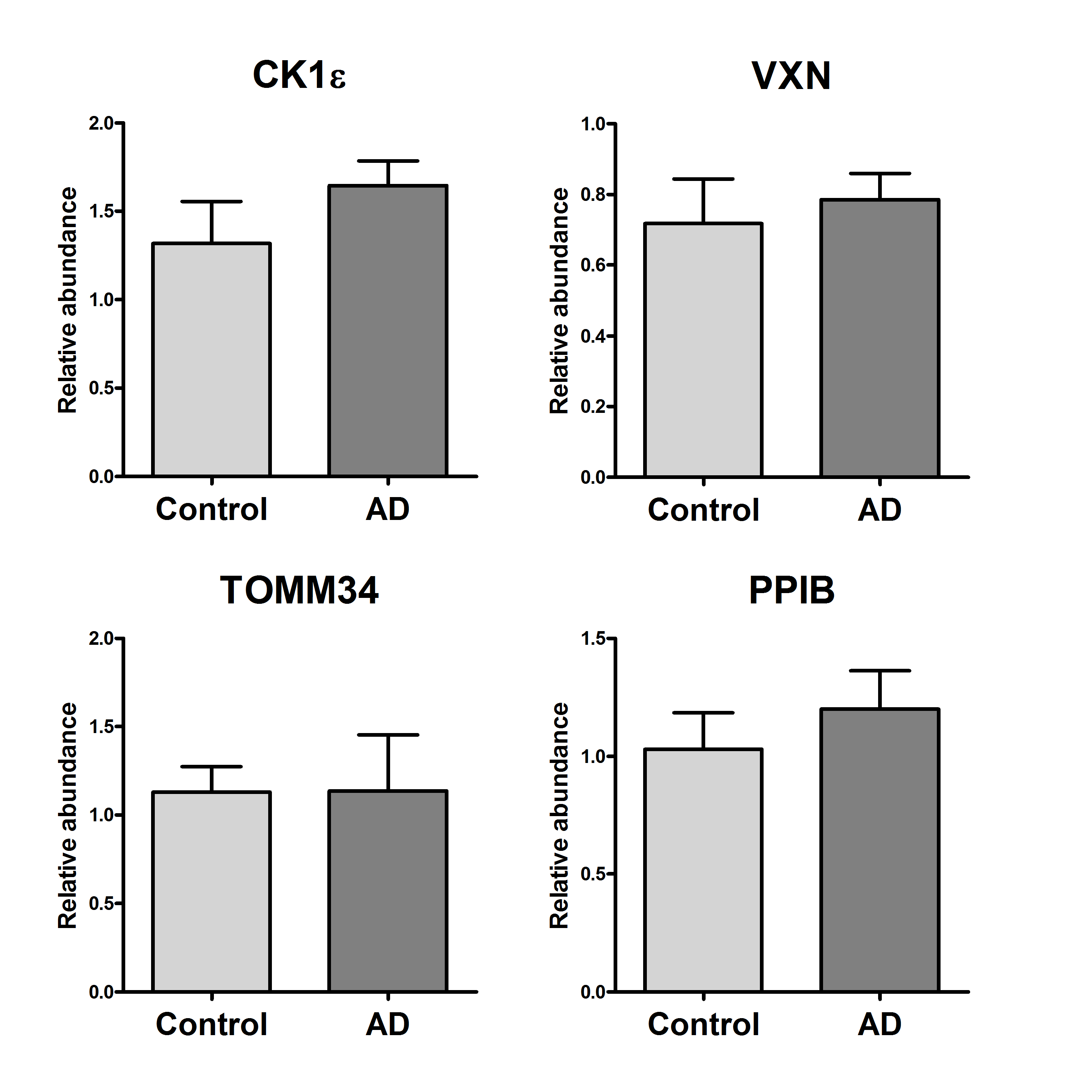

Supplement: Supplementary file 5 — Supplementary file5 (TIF 25632 KB) [file 401_2020_2261_MOESM5_ESM.tif]
